# Electrospun Biodegradable Poly(L-lactic acid) Nanofiber Membranes as Highly Porous Oil Sorbent Nanomaterials

**DOI:** 10.3390/nano12152670

**Published:** 2022-08-03

**Authors:** Jizhen Yang, Fan Li, Guibin Lu, Yuanbin Lu, Chuanbo Song, Rong Zhou, Shaohua Wu

**Affiliations:** 1 College of Textiles & Clothing, Qingdao University, Qingdao 266000, China; qq1132419018@163.com (J.Y.); qianzhan0416@163.com (F.L.); shaohua.wu@qdu.edu.cn (S.W.); 2 Shandong Xingguo Xinli Environmental Protection Co., Ltd., Zibo 255000, China; luguibin011@126.com (G.L.); luyuanbin7932@163.com (Y.L.); 18678155215@163.com (C.S.)

**Keywords:** PLLA, electrospinning, porous structure, acetone post-treatment, oil absorption

## Abstract

Crude oil spills seriously harm the ocean environment and endanger the health of various animals and plants. In the present study, a totally biodegradable polymer, poly(L-lactic acid) (PLLA), was employed to fabricate highly porous oil absorbent nanofibrous materials by using a combination of electrospinning technique and subsequent acetone treatment. We systematically investigated how the electrospinning parameters affected formation of the porous structure of PLLA nanofibers and demonstrated that PLLA nanofibers with decreased and uniform diameter and improved porosity could be rapidly prepared by adjusting solution parameters and spinning parameters. We also demonstrated that the acetone treatment could obviously enhance the pore diameter and specific surface area of as-optimized electrospun PLLA nanofibers. The acetone treatment could also improve the hydrophobic property of as-treated PLLA nanofiber membranes. All these led to a significant increase in oil absorption performance. Through our research, it was found that the oil absorption of PLLA nanofiber membrane increased by more than double after being treated with acetone and the oil retention rate was also improved slightly.

## 1. Introduction

Petroleum plays an important role in many industries [1]. The large demand for petroleum from various countries has promoted the rapid development of the petroleum transportation industry [2]. In the course of transportation, vessel failure or other accidents may lead to oil spills [3]. Oil spills in waterways can pollute groundwater and freshwater supplies in cities and cause long-term damage to fisheries and wildlife [4]. Oil spills account for about half of total ocean pollution, causing fatal damage to marine life and marine ecology [5,6]. Over the past few decades, many large-scale oil spills have occurred in countries around the world, which has raised the public’s awareness of the risks of oil spills in most parts of the world [7,8]. The frequency of oil spills is extremely harmful, including the pollution of streams and rivers, the destruction of forests, and the loss of biodiversity, as well as negative socio-economic impacts [9,10].

In recent years, a variety of polymers have been used in the field of oil–water separation, such as polypropylene cyanide, polyester materials, and polypropylene [11,12,13]. Researchers can give these polymers certain post-treatments to obtain good oil absorption capacity. However, these polymers are not environmentally friendly oil-absorbing materials due to their lack of degradation properties. Therefore, it is of great significance to study environmentally friendly and practical oil-absorbing material for environmental protection and green and sustainable development.

Poly(lactic acid) is biodegradable and easy to treat. It is a kind of green and environment-friendly material [14,15]. It has been widely used in filtration, adsorption of heavy metal ions, and biomedicine [16,17,18]. Because of the great harms caused by oil leakage, research into oil-absorbing materials is a hot topic [19]. Polylactic acid (PLA) is also favored by researchers in terms of oil absorption due to its excellent superhydrophobic properties and environmental protection characteristics, and the oil–water separation performance of PLA-based materials can be further enhanced by the selection of appropriate forming process and post-treatment strategies [20,21,22,23,24].

Electrospinning is a common method for the preparation of nanofibers. It is popular due to its simple device, low cost, and controllable process [25,26]. Porous nanofiber prepared by electrospinning have the advantages of high specific surface area, high porosity, and high adsorption [27]. It is widely used in filtration, sensors, and adsorption [28,29]. A variety of polymers such as polycaprolactone, polystyrene, and PLA can be obtained by electrostatic spinning [30,31,32,33]. During electrospinning, the solvent is more volatile than the non-solvent, so the jet forms a different phase separation structure, and the further evaporation of the residual non-solvent will lead to the formation of porous fibers [20,22,34,35]. Electrospun porous PLA nanofibers can usually be obtained by two methods: one employs the phase separation phenomenon during spinning, and the other employs the selection of appropriate post-treatment strategies [17,21,36]. Up to now, some existing studies have been conducted to investigate the structure and properties of electrospun porous PLA nanofibers [37,38]. In addition, porous PLA fibers can also be prepared by some advanced electrospinning techniques such as blending spinning or coaxial spinning [39,40]. Moreover, several existing studies indicated the acetone post-treatment seemed to be an effective strategy for the increase of pore number and specific surface area of PLA fibers [17,41]. However, how to generate porous PLA nanofibers with decreased and uniform diameter and improved pore size and porosity still remains a huge challenge. Moreover, how to simplify process flow is also of significant importance, because ideal PLLA porous nanofibers cannot be spun easily by a simple phase-separation technique.

In this study, a simple double-solvent system was employed to dissolve PLLA polymer, and a conventional electrospinning process was employed to generate porous PLLA nanofibers using the phase-separation theory. The influence of the solution parameters and spinning parameters on the generation of porous PLLA nanofibers with ideal morphology and structure was systematically investigated. Moreover, an acetone post-treatment strategy was further applied to the as-optimized electrospun porous PLLA nanofibers, which was expected to improve the structure and performance of porous PLLA nanofibers. To verify our hypothesis, the adsorption properties of as-generated different PLLA nanofibers to silicone oil, soybean oil, and machine oil, were investigated and compared. This study aims to provide meaningful reference and guidance for the advanced development of biodegradable PLLA nanofibers using a simple fabrication strategy as ideal oil–water separation material.

## 2. Materials and Experimental Methods

### 2.1. Materials

PLLA (product number: 6202D, with ~2% D-lactic acid) was obtained from Nature-Works LLC, Blair, NE, USA. The weight-average molecular weight was 1.17 × 10^5^ Da, and the polydispersity index was 2.00. Dichloromethane (DCM) and dimethylformamide (DMF) were purchased from Shanghai Aladdin Biochemical Technology Co., Ltd., Shanghai, China. Acetone was purchased from Sinopharm Chemical Reagent Co., Ltd., Shanghai, China. Soybean oil was bought from Yihai Kerry Grain, Oil and Food Co., Ltd., Qingdao, China. Silicone oil (H201-100) was provided by Sinopharm Chemical Reagent Co., Ltd., Shanghai, China. Machine oil (5W-40 Mobil No.1) was provided by Exxon Mobil Corporation, Taicang, China.

### 2.2. Sample Preparation

**Figure 1:**
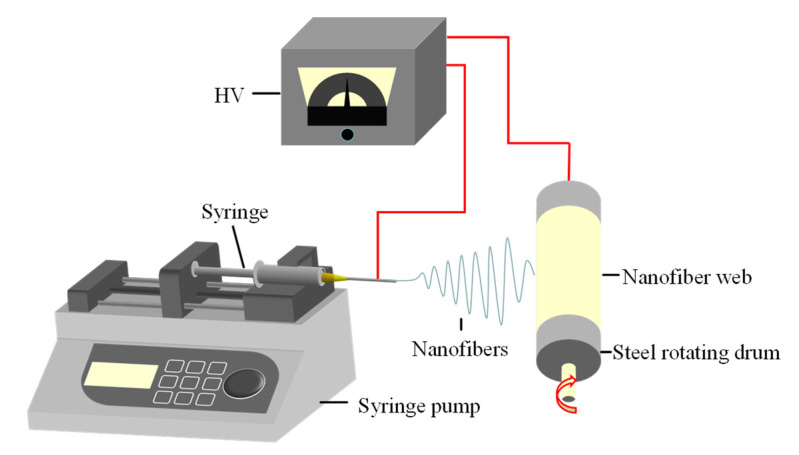
Schematic diagram of electrospinning setup.

We dissolved PLLA particles in a dual-solvent system consisting of DCM and DMF to obtain the spinning solution. Then, the spinning solution was poured into a syringe with a metal needle (1 mm in diameter) and the solution feeding rate controlled by the injection pump was set at 1.0 mL/h. The electrospinning process was performed at a temperature of 25 ± 2 °C and relative humidity of 50 ± 5%. The as-generated nanofibers prepared by electrospinning were collected on a metal drum. Finally, the resulting fiber film was dried in an oven at 60 °C for 24 h. This electrospinning setup is shown in Figure 1.

The concentration of the spinning solution (8–14%), the ratio of the two solvents (DCM/DMF: 10/0-7/3), and the voltage (14–20 kV) and receiving distance (14–20 cm) during spinning were adjusted to explore the effect of these parameters on the surface pores of the fiber. We collected the PLLA porous nanofiber membrane under the optimal parameters of the holes, and it was soaked in 90% acetone solution at room temperature for 5 min and then dried in air.

### 2.3. Characterization

The morphology and structure of the nanofiber membrane were investigated by scanning electron microscopy (SEM, Phenom Pro SEM, Hitzacker, Germany). The samples were subjected to gold sputter-coating in a vacuum prior to imaging (SBC-12, Beijing KYKY Technology Co., Ltd., Beijing, China). The distribution of fiber diameter was determined by Nano Measurer software (Nano Measurer 1.2, Fudan University, Shanghai, China). The irregular fiber morphology made it difficult to measure the fiber diameter, and we hypothesized that the fibers have nearly circle-like cross-sections. To improve the testing accuracy, more than 100 different locations were randomly chosen based on 3 different SEM images for each specimen. The structures and the elemental analysis of the nanofiber membrane were characterized by a Fourier-transform infrared spectroscope (FT-IR, Nicolet iS10, Thermo Fisher Scientific, Waltham, MA, USA).

The pore size distribution and average pore sizes were measured according to the bubble method using aperture tester (TOPAS PSM-165, Frankfurt, Germany). All data were tested in at least 5 sets of samples and averaged. The specific surface area and pore size of the sample fibers were characterized by the automatic specific surface area analyzer (Micromeritics, Atlanta, GA, USA). The Brunauer−Emmett−Teller (BET) and density functional theory (DFT) methods were used to calculate specific surface area and pore size distribution.

The contact angle was measured on a contact angle goniometer (JY-PHb), a drop of the ionized water was placed on the membrane, and the CA values were recorded after the water drop was kept on the surface of the nanofiber membrane for 60 s. The average CA values were obtained by testing the same sample at five different positions. The mechanical property of the nanofiber membrane was tested using the universal tensile testing machine (Instron 6025, Jinan Liangong Testing Technology Co., Ltd., Jinan, China), the sample size was 20 mm × 70 mm, the distance between the two clamps was 50 mm. Each group of five samples was tested and averaged.

### 2.4. Oil Absorption Performance Test

PLLA nanofiber film of a certain quality was immersed into three beakers containing soybean oil, corn oil, and diesel oil, respectively. It was taken out every 30 min by the hanging angle method. After dropping freely for 5 min, the fiber film after oil absorption was weighed, and the oil absorption ratio was called *Q*. The experiment was repeated five times and averaged. *Q* was calculated according to the equation:

$$Q=\frac{W_{1}-W_{0}}{W_{0}}$$

where *Q* is the oil absorption rate (g/g), *W*_0_ and *W*_1_ are the mass of PLLA fiber membrane before and after oil absorption respectively.

The PLLA fiber membrane after saturated oil absorption was placed on the copper network and weighed after natural dropping for 1 h. The ratio of the mass of the PLLA fiber membrane after natural dropping for 1 h to the mass after saturated oil absorption is recorded as the oil retention Rate *K*.

$$K=\frac{W_{2}}{W_{1}}\times 100\%$$

where *K* is the oil retention rate, *W*_1_ and *W*_2_ are the mass of PLLA nanofiber membrane after saturated oil absorption and 1h after natural droplet dropping.

## 3. Results and Discussion

During electrospinning, the morphology of the fibers is affected by numerous factors, such as: solution concentration, solvent ratio, solution viscosity, temperature, humidity and so on. In this study, the influence of solution concentration, solvent ratio, electric field intensity, voltage, and distance on fiber morphology was explored.

### 3.1. The Influence of Solution Concentration on Fiber Morphology and Pores

**Figure 2:**
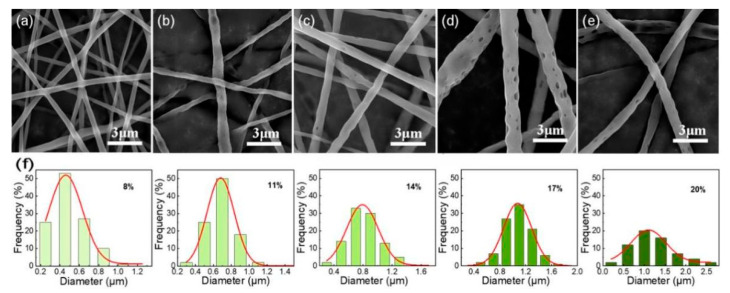
SEM images of the electrospun fibrous membrane with different PLLA concentrations (wt%), (**a**) 8%; (**b**) 11%; (**c**) 14%; (**d**) 17%; (**e**) 20%; (**f**) Gaussian fitting curve of fiber diameter distribution prepared under different solutions.

When studying the influence of PLLA concentration on fiber morphology, other spinning parameters were fixed. The results show that as the PLLA concentration increases, the fiber diameter becomes larger. Although the fiber diameter is evenly distributed when the solution concentration is low (8 and 11%), no holes are formed on the fiber surface (Figure 2a,b,f). This is because the lower the concentration of the spinning solution, the easier it is for the fibers to be stretched and stretched thinner, and the porous structure is less easily formed. Figure 2c,d,f shows the uniformity of fiber diameter distribution becomes worse for higher solution concentrations (14 and 17%), but obvious pore structure appears on the surface of the fiber. The pores on the surface of the fiber with a concentration of 17% are denser and deeper than those with a concentration of 14%, which reveals that only a proper concentration of the spinning solution can prepare porous PLLA ultrafine fibers. As the concentration of the solution increases further, the spinning liquid concentration or viscosity is too high, and the PLLA chains are highly entangled and thus not easily stretched in the process of filament formation, and the solvent cannot be evaporated in time, leading to insufficient phase-change separation. As a result, the fiber diameter uniformity is poor and the holes formed are very small and sparse (Figure 2e,f).

### 3.2. Influence of Solvent Volume Ratio on Fiber Morphology and Pores

**Figure 3:**
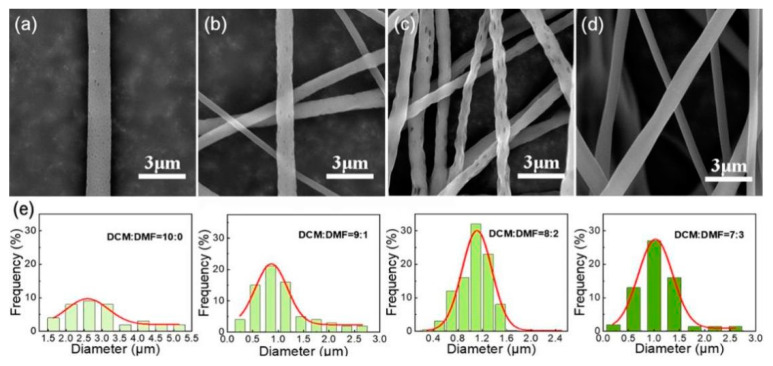
SEM images of the electrospun fibrous membrane with different solvent ratios, (**a**) DCM:DMF = 10:0; (**b**) DCM:DMF = 9:1; (**c**) DCM:DMF = 8:2; (**d**) DCM:DMF = 7:3; (**e**) Gaussian fitting curve of fiber diameter distribution prepared under different solvent ratios.

Fixing other spinning parameters, the volume ratio of benign solvent DCM and non-benign solvent DMF was changed to explore the influence of the solvent ratio on fiber morphology and holes. As shown in Figure 3a,e, the fiber received with only DCM single solvent has a large diameter and very poor uniformity. Because DCM is very volatile, when it is used as a solvent alone for spinning, the nozzle is likely to be blocked, spinning cannot proceed smoothly. The DCM/DMF ratio was set to 8:2 or 9:1, Figure 3b,c,e displays that there are holes on the fiber surface, however, when the solvent ratio is 8:2, the fibers prepared are larger in diameter and more evenly distributed than when the solvent ratio is 9:1. Because the increase of DCM content reduced the viscosity of the solution, and the jet flow is easier to be drawn and thinned. When the DCM/DMF volume ratio is 7:3, although the fiber is more evenly distributed, there was no pore structure on its surface (Figure 3d,e). In summary, the pore structure on the fiber surface is greatly affected by the mixed solvent DCM/DMF volume ratio. For the DCM/DMF ratio of 6:4, the spinning solution becomes turbid during the dissolution process, phase separation occurs, and spinning cannot be performed.

### 3.3. Influence of Voltage and Curing Distance on Fiber Morphology and Porosity

**Figure 4:**
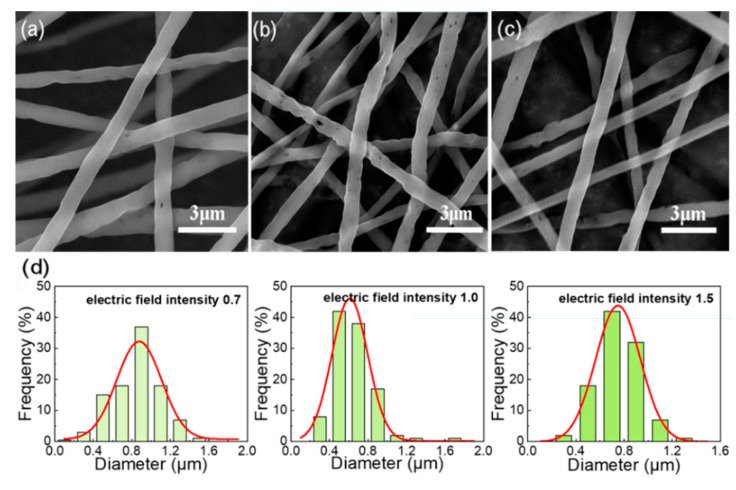
SEM images of the electrospun fibrous membrane with 14% solution concentration at different voltage, (**a**) 11 kV; (**b**) 16 kV; (**c**) 24 kV; (**d**) Gaussian fitting curve of fiber diameter distribution prepared under different voltages.

The solution concentration and curing distance were set as 14% and 16 cm, respectively, and the other spinning conditions were fixed. The voltage was changed to observe the effect of electric field intensity (voltage/curing distance) on the formation of holes on the fiber surface. As can be seen from Figure 4, under higher electric field intensity, the fibers are rapidly stretched to the collecting plate, the solvent volatilization time is too short to the pore structure can be formed. At low electric field intensity, the fiber surface has almost no holes. The formation of large and dense holes on the surface of the fiber can occur only when the electric field intensity is moderate (voltage/curing distance = 1.0).

**Figure 5:**
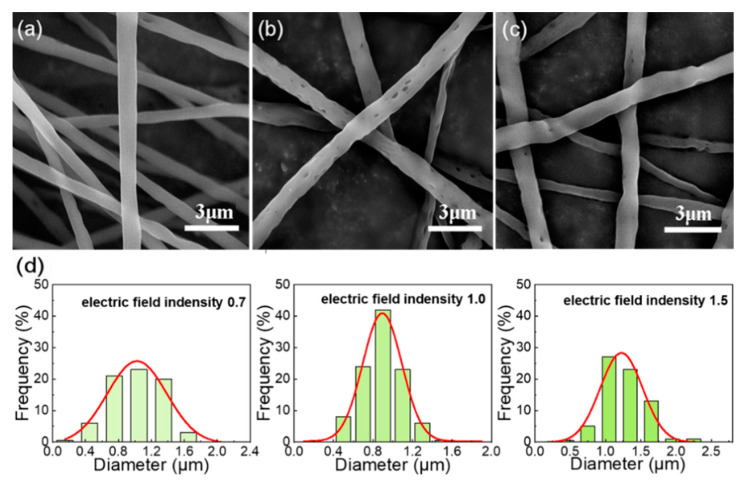
SEM images of the electrospun fibrous membrane with 17% solution concentration at different curing distance, (**a**) 22 cm; (**b**) 16 cm; (**c**) 11 cm; (**d**) Gaussian fitting curve of fiber diameter distribution prepared under different curing distances.

The solution concentration and voltage were set as 17% and 16 kV, respectively, and the other spinning conditions were fixed. The curing distance was changed to explore the effect of electric field intensity on the fiber and its surface holes. The same result as when changing the voltage is obtained. These results provide important insights into the more perfect hole structure is obtained under the condition that the voltage and curing distance ratio is 1 (Figure 5).

**Figure 6:**
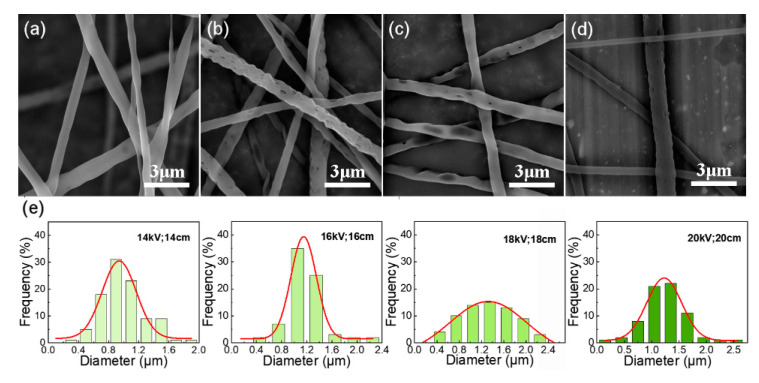
SEM images of the electrospun fibrous membrane with different voltage and collecting distance, (**a**) 14 kV, 14 cm; (**b**) 16 kV, 16 cm; (**c**) 18 kV, 18 cm; (**d**) 20 kV, 20 cm; (**e**) Gaussian fitting curve of fiber diameter distribution prepared under different voltages and collecting distances.

Other spinning conditions remain the same, and the electric field intensity (voltage/curing distance) was set to 1 to study the effect of voltage and curing distance on fiber morphology and surface pores. From Figure 6a we can see that no hole structure is formed on the fiber surface when the voltage and distance are 14 kV and 14 cm, this is caused by the fact that the receiving distance is so short that the solvent fails to volatilize and is received at the receiving device. If the receiving distance is too far during spinning, the solution jet easily becomes unstable. Therefore, when the voltage and distance are 18 kV and 18 cm or 20 kV and 20 cm, there are sparse and small holes on the fiber surface, and the fiber diameter distribution is extremely uneven (Figure 6c–e). The results obtained from the correlational analysis of the fibers with uniform diameter and dense surface pore distribution were successfully prepared under suitable spinning voltage and curing distance (16 kV, 16 cm) in Figure 6b,e.

**Figure 7:**
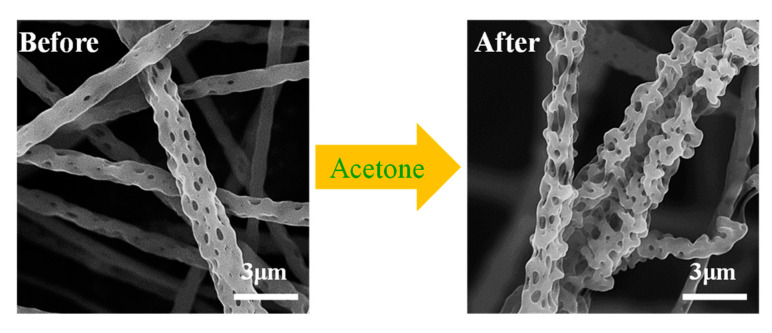
The changes of pores on the fiber surface before and after acetone treatment.

As shown in Figure 7, after the fiber membrane was post-treated with acetone, the fiber morphology changed greatly. It can be observed that the surface of the fiber becomes rougher and has more holes. More holes can capture more oil molecules when absorbing oil, and the rough fiber surface helps to lock the oil molecules and improve the oil retention rate.

### 3.4. FTIR Analysis

**Figure 8:**
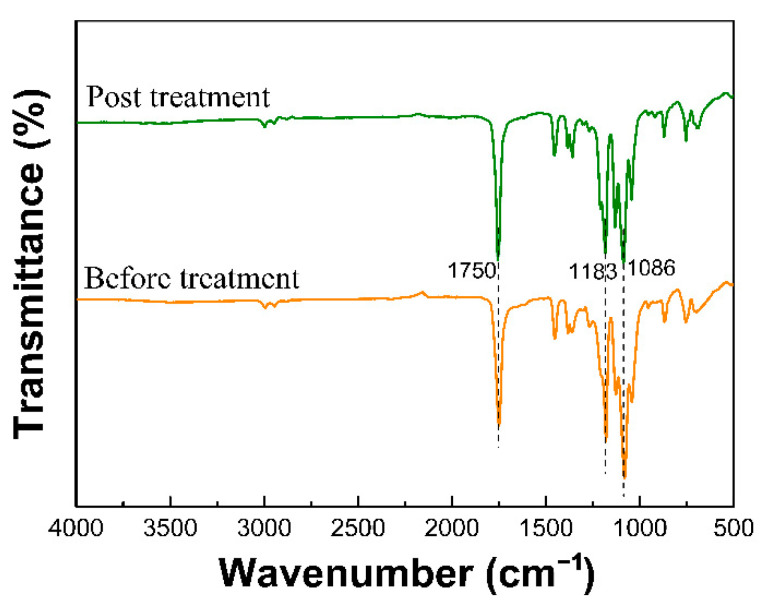
The ATR-FTIR spectra of samples before and after treatment with acetone.

PLLA material has good oil absorption performance due to ester group hydrophobic and oil-wet properties. As shown in Figure 8, the peak at 1750 cm^−1^ represents C=O stretching vibration, and there are two absorption bands within the range of 1300–1000 cm^−1^, which represent the C–O–C asymmetric stretching vibration of the ester. After acetone treatment, the specific surface area of the PLLA nanofiber membrane was increased without any damage to its functional groups.

### 3.5. Wettability

**Figure 9:**
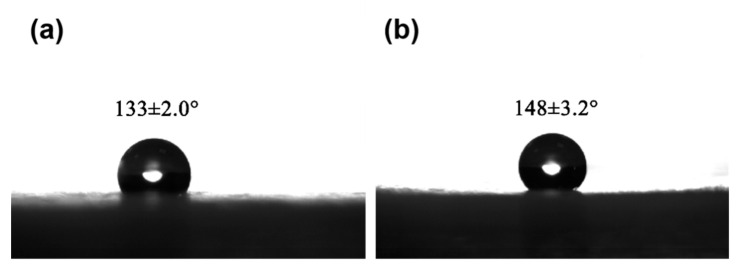
Water contact angle measurement of PLLA fiber membrane: (**a**) before acetone treatment; (**b**) after acetone treatment.

The water contact angle was measured for the fiber membranes before and after acetone treatment, and the results were presented in Figure 9. It is well known that when the water contact angle of a membrane is greater than 90°, it indicates that the membrane is hydrophobic. It can be observed that the water contact angle of PLLA fiber membranes is greater than 130° regardless of acetone treatment, indicating that PLLA has good hydrophobicity. This is consistent with the results of the infrared proof of the ester group in Figure 8. Many existing studies demonstrated that water contact angle of a membrane is closely related to the surface morphology and roughness, and the hydrophobicity of a membrane increased with the surface roughness [20,42,43]. Our present study also found that the contact angle of the acetone-treated membrane is larger than that of the untreated one, because the acetone treatment can significantly improve the surface roughness of PLLA fiber membrane. Therefore, the enhanced hydrophobicity makes the acetone-treated membrane exhibit better oil absorption performance.

### 3.6. Mechanical Properties

**Figure 10:**
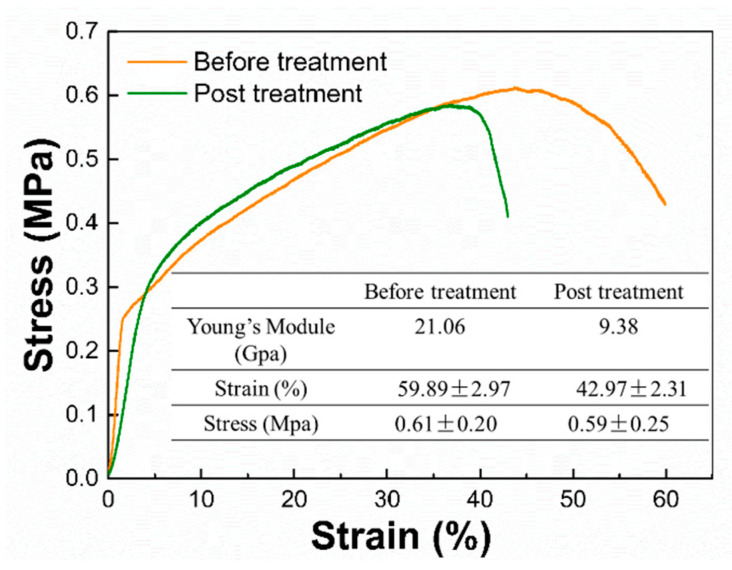
Stress−strain curves of the of PLLA fibrous membranes before and after post-treatment.

From the data in Figure 10, it is obvious that the mechanical properties of the membrane treated with acetone are worse. After post-treatment, the strain of the PLLA nanofiber membrane decreases greatly, the elastic modulus decreases, the flexibility of the membrane increases, and the membrane is more prone to deformation. This indicates that, after treatment, there is certain damage to the mechanical properties of the fiber membrane, which may be caused by the low molecular weight of PLLA, but does not affect its oil absorption performance.

### 3.7. BET Analysis and Pore Size Analysis of Fiber Membrane

**Figure 11:**
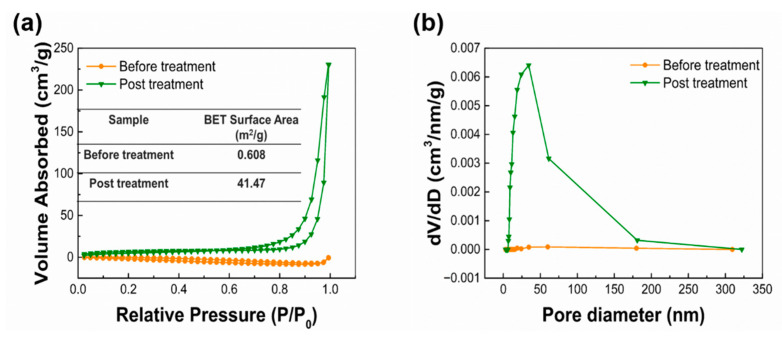
(**a**) N_2_ adsorption-desorption isotherm graphs of PLLA fibers before and after acetone treatment. (**b**) Pore diameter distribution curves of PLLA fibers before and after acetone treatment.

N_2_ adsorption analysis was performed on the fiber membrane before and after acetone treatment, The obtained isotherms are shown in Figure 11a. It is obvious that the adsorption curve and desorption curve of the isotherm is inconsistent, and hysteresis loops can be observed. According to IUPAC separation, the PLLA nanofiber membrane belongs to type IV isotherm. As can be seen from the table in Figure 11a, the specific surface area of the fiber membrane increased significantly after acetone treatment, which was caused by a large increase in the number of holes on the fiber. The increase of the specific surface area of fiber film is beneficial to improve its oil absorption performance. The BJH method was used to analyze the pore size distribution on the fiber. As shown in Figure 11b, the pore size of the sample treated with acetone is much larger than that of the sample not treated with acetone, and most of the pore size is below 50 nm, which could be classified as mesopore. The sample without acetone treatment has a large stomatal diameter and low content, which is not conducive to the adsorption of oil molecules.

### 3.8. Oil Absorption

**Figure 12:**
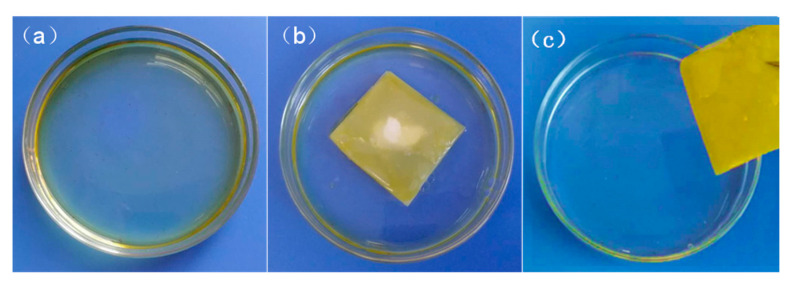
Oil absorption process of PLLA nanofiber membrane with acetone treatment: (**a**) before oil absorption; (**b**) floating on the water; (**c**) after oil absorption.

The acetone-treated PLLA fiber membrane and the untreated film were immersed in three different types of oil products, respectively, and the oil absorption ratio and oil retention rate of the two fiber films for different oil products were calculated and compared. The oil absorption process of PLLA nanofiber membrane with acetone treatment is shown in Figure 12.

**Figure 13:**
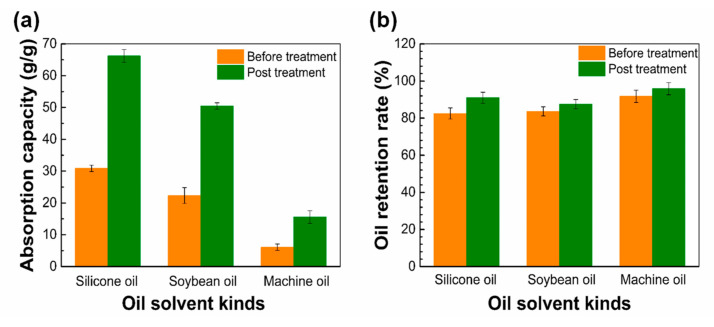
(**a**) Oil absorption ratio of fiber membrane before and after acetone treatment; (**b**) Oil retention rate of fiber film before and after acetone treatment.

Results are shown in Figure 13. Obviously, from Figure 13a, it can be seen that the PLLA fiber membrane without acetone post-treatment has oil absorption ratios of 30.86, 22.34 and 6.08 g/g for silicone oil, soybean oil, and motor oil, respectively. After acetone treatment, the oil absorption rate of the three oils was almost doubled. This is because the post-treatment causes the fiber surface has more holes, which increases the specific surface area of the fiber membrane and causes it to have better oil absorption performance. It can be observed from Figure 13b that the PLLA fiber membrane has a strong oil retention rate, which can reach above 80% for all the three tested oil products, and the oil retention rate can be slightly improved by acetone post-treatment.

## 4. Conclusions

In this study, the concentration of PLLA in the spinning solution, the ratio of the two solvents, the voltage, and the receiving distance during spinning were adjusted to find the optimal parameters for preparing the porous nanofiber membrane. The prepared fiber film was post-treated with acetone for oil absorption, which causes the oil absorption efficiency of the fiber film to at least double. These results indicate that polylactic acid produced by electrostatic spinning is a potential environment-friendly oil-absorbing material after being treated with acetone, and it can be applied in the absorbing field.

## Data Availability

Data is contained within the article.
